# Nocturnal heart rate rising is a risk factor for poor renal outcomes in patients with chronic kidney disease and hypertension

**DOI:** 10.1111/jch.14428

**Published:** 2022-02-07

**Authors:** Xiang Liu, Huan Zhou, Gen Li, Fangming Li, Lingqiu Dong, Siqing Wang, Zheng Jiang, Jiaxing Tan, Aiya Qin, Yi Tang, Wei Qin

**Affiliations:** ^1^ Division of Nephrology, Department of Medicine, West China Hospital Sichuan University Chengdu Sichuan China; ^2^ West China school of Medicine Sichuan University Chengdu Sichuan China; ^3^ Division of Nephrology Department of Medicine Chengdu 7th People's Hospital Chengdu Sichuan China

**Keywords:** ambulatory blood pressure monitoring, CKD, HR non‐dippers, HR risers, renal outcomes

## Abstract

The association of heart rate (HR) dipping pattern with renal outcomes in chronic kidney disease (CKD) patients with hypertension has never been investigated. In order to demonstrate if HR dipping pattern is a risk factor for renal outcomes, cardiovascular (CV) diseases, and mortality in hypertensive patients with CKD, we conducted the prospective longitudinal observational study. Patients were divided into three groups according to their nocturnal HR: HR dippers (night–day HR ratio ≤ 0.9), HR non‐dippers (0.9 < night–day HR ratio ≤ 1.0), and HR risers (night–day HR ratio > 1.0). The primary outcome was renal endpoint, a composite outcome of progression to end‐stage renal disease (ESRD) or estimated glomerular filtration rate (eGFR) decline ≥ 50%; the secondary outcomes included poor renal outcomes, CV events, and death. A total of 34 (11.3%) patients reached renal endpoint after a follow‐up of 34 ± 17 months. Both HR non‐dippers and HR risers were predictive to renal endpoint (hazard ratio 2.58, 95% confidence interval (CI) 1.04‐ 6.4, *P* = .04; hazard ratio 3.95, 95% CI 1.33‐ 11.79, *P* = .01, respectively), while only HR risers was shown to be correlated with a decline in eGFR≥ 50% (hazard ratio 5.28, 95% CI 1.45–19.16, *P* < .05), and decline in eGFR (β ‐0.17, 95% CI ‐0.33‐ ‐0.01, *P* = .04). No predictive value was found for HR dipping pattern to mortality and CV events. In conclusion, our study provided the first evidence that HR non‐dippers, especially risers were a risk factor for poor renal outcomes in hypertensive patients with CKD.

## 1 INTRODUCTION

Chronic kidney disease (CKD) is a public health problem, with a 3–18% prevalence in the general population.^1^ It has caused a great global burden owing to the high risk of advancing to end‐stage renal disease (ESRD), and cardiovascular (CV) complications.^1^, ^2^, ^3^ It is vitally imperative to find prognostic factors to help identify early intervention strategies to slow the progression of CKD and improve prognosis of renal diseases.

Heart rate (HR) is a clinically accessible indicator of vital signs and sympathetic activity,^4^ which has been reported to be predictive of all‐cause mortality, cardiovascular disease, and renal outcome.^5^, ^6^, ^7^ Similar to blood pressure, HR follows circadian rhythms; that is, it normally decreases by 10–20% at night due to the effect of the parasympathetic system,^8^, ^9^ a night HR decline less than 10% from day is defined as the HR non‐dippers.^10^

Circadian rhythms of heart rate can be obtained by ambulatory blood pressure monitoring(ABPM), and has been recommended to be applied in hypertensive patients by guidelines, because of the better predictability of blood pressure (BP) parameters from ABPM to prognosis compared to office/home BP.^11^, ^12^, ^13^

HR non‐dippers has been reported to be associated with all‐cause mortality at the end of the 1990s by Verdecchia and associates,^14^ this was later confirmed by a few studies on general or hypertensive patients, which showed that HR non‐dippers was related to preclinical cardiac damage, mortality, and CV events.^6^, ^15^, ^16^, ^17^, ^18^ However, to the best of our knowledge, studies on the association of HR dipping pattern with prognosis in CKD patients are very limited. The first study related to HR dipping pattern in CKD patients occurred in 2019, which reported the high prevalence (43.3% in the hypertensive pre‐dialysis patients and 77.4% in the hypertensive hemodialysis patients) of non‐dipping HR in CKD patients^19^; subsequently, a study conducted by Cui and associates in 2021 demonstrated that non‐dipping HR was a prognostic marker of all‐cause mortality in CKD patients with stage 5.^20^

To further investigate the association of HR dipping pattern with renal and heart outcomes in patients with CKD and hypertension, we conducted this prospective longitudinal observational study.

## 2 METHODS

### 2.1 Patients and clinical data

This was a prospective longitudinal observational study including patients from two centers (West China Hospital of Sichuan University and Chengdu 7th People's Hospital) in China. Adult patients were eligible if they: (1) had chronic kidney disease, (2) have been diagnosed with hypertension in the clinic, and (3) agreed to take 24h ABPM. Exclusion criteria were patients with 1) eGFR < 15 mL/min per 1.73 m^2^ or dialysis; (2) history of malignancy, and (3) kidney transplant. Patients with albuminuria (albumin/creatinine ratio ≥30 mg/g), or the estimated glomerular filtration rate (eGFR) < 60 mL/min / 1.73 m^2^, or abnormalities of kidney structure for over 3 months were diagnosed with CKD.^21^ The eGFR value was estimated from serum creatinine levels with Chronic Kidney Disease Epidemiology Collaboration (CKD‐EPI) equation.^22^ The study protocol was approved by the ethics committee of the West China Hospital, Sichuan University, and was approved by the Institutional Review Board (http://www.ClinicalTrials.gov; TCTR20180313004). Written informed consent was obtained from all patients.

Patients were collected from January 2014 to December 2019, a total of 304 CKD patients met the inclusion /exclusion criteria and completed the 24h ABPM. According to their nocturnal HR from 24h ABPM, the participants were divided into 3 groups, including HR dippers (night–day HR ratio ≤0.9), HR non‐dippers (0.9 < night–day HR ratio ≤1.0), and HR risers (night–day HR ratio > 1.0). Demographic data (age, gender, body mass index (BMI)), Medical history (current alcohol, current smoking, CV history, antihypertensive drugs, diabetes mellitus (DM)), and laboratory tests (biochemical parameters, urinary protein test) were recorded at baseline. Patients were followed up by checking the electronic databases in the hospital and phone calls; the follow up was from the day of ABP, until December, 2020, death, or CKD progression, and censored on the date they had the last clinic visit or phone answering. Only patients with follow‐up time for more than six months were included into the final analysis.

### 2.2 Blood pressure and heart rate measurements

Office BP and HR were measured by experienced nurses at the time that the participants were admitted to our hospital; the mean value of three consecutive measurements at 5‐minute intervals with a mercury sphygmomanometer after patients rest quietly for 5 to 10 minutes was recorded. The twenty‐four‐hour ambulatory blood pressure (ABP) and heart rate monitoring was performed via The Space Labs 90217 device (Space Labs Medical, Redmond, Washington, USA) in West China Hospital and ABPM 6100 (Welch Allyn, USA) in Chengdu 7th People's Hospital, with BP and HR readings set at 20‐minute intervals from 6:00 am to 10:00 pm, and 30‐minute or 60‐minute intervals from 10:00 pm to 6:00 am. The BP and HR of daytime, nighttime was defined as the mean values during the period from 6:00 am to 10:00 pm and 10:00 pm to 6:00 am, respectively; patients were instructed to take their usual activities and receive antihypertensive drugs as usual, and was encouraged to sleep no later than 10:00 pm, and get up at nearly 6:00 am. A measurement with at least 70% of diurnal and nocturnal BP and HR readings was regarded as a successful ABP. The ABP must be taken within three days after the measurement of office BP. Both office BP and ABP measurements were taken from the non‐dominant arm with an appropriate cuff size based on arm circumference at the time of enrollment.

### 2.3 Definitions

Dipping pattern of BP and HR were calculated with the formula: mean night‐day ratio of systolic blood pressure (SBP), diastolic blood pressure (DBP) and HR, patients were diagnosed with normal BP or HR dippers if the night‐day ratio was ≤0.9, or non‐dippers if the ratio was > 0.9 and ≤1.0,^10^, ^23^ or risers if the ratio was > 1.0. HR non‐dipping pattern included HR non‐dippers and risers. Patients were defined at goal for ABP when 24 hours, daytime and nighttime BP was < 130/80, < 135/85, and < 120/70 mmHg, respectively, and at goal for office BP if < 140/90 mmHg.^24^

### 2.4 Outcomes

The primary outcome was a renal endpoint, which was defined as a composite of progression to ESRD (eGFR < 15 mL/min.1.73 m^2^ or dialysis), or eGFR decline ≥ 50%. The secondary outcome included (1) death; (2) deterioration of kidney function, including a composite of progression to ESRD, eGFR decline ≥ 30%; and individual components of the composite kidney outcomes; (3) the rate of change of eGFR from baseline, calculated as (eGFR at endpoint ‐ eGFR at baseline)/ eGFR at baseline; (4) CV events, which was defined as a fatal or nonfatal newly occurred CV disease, including coronary heart disease, myocardial infarction, heart failure, stroke, angina pectoris, whichever occurred first. Patients were followed up every 6 months by checking the electronic databases in the hospital and phone calls, mainly including disease progression and laboratory tests (biochemical parameters, urinary protein test); the follow‐up was from the day of ABP, until December 2020, death, or renal endpoints, and censored on the date they had the last clinic visit or phone answering.

### 2.5 Statistical analysis

Statistical analysis was performed using IBM SPSS Statistics for Windows, Version 20.0 (IBM Corp, Armonk, New York, USA). All data were expressed as mean ± standard deviation (SD) for normally distributed data, median values with their interquartile range for skewed data, and numbers (n) with percentage (%) for categorical variables. Data were analyzed by using the Chi‐square test or Fisher's exact test for categorical variables, Student's t‐test for normally distributed data, and Wilcoxon rank‐sum test for continuous skewed variables.

Kaplan‐Meier survival function estimates, the log‐rank test was applied to compare the incidence rates of events in different groups; Multivariable Cox regression model was used to investigate associations of HR dipping pattern with renal and CV outcomes. The proportional hazards (PH) assumption was tested by assessing the log‐minus‐log plots of survival. The multivariate linear regression model was applied to detect the association of HR dipping pattern with the rate of change of eGFR from baseline, the regression ß coefficient represented the contribution of the independent variables to the dependent variables. Hazard Ratios, 95% CIs (CIs) were calculated and a two‐tailed *P* value < .05 was considered statistically significant.

## 3 RESULTS

### 3.1 Baseline characteristics

Two (0.66%) of the included 304 patients at baseline were lost to follow up, finally, 302 hypertensive participants with CKD and good quality ambulatory BP/HR recordings (> 70% valid recordings) were analyzed (44% men, mean age 72 ± 11years, BMI 23.4 ± 4.4 kg/ m^2^); 48 (15.9%), 45 (14.9%) patients were a current smoker or drinker, respectively. One hundred twenty‐two (40.4%) patients had a CV history, 82 (27.2%) of which had arrythmia, and 218 (72.2%) were complicated with diabetes mellitus (DM).

The prevalence of HR non‐dippers and risers was 64.2%, including 152 (50.3%) non dippers and 42 (13.9%) risers. Table 1 shows the baseline clinical characteristics of the study population divided by HR dipping pattern. Compared to HR dippers, patients with HR non‐dippers or risers tended to be older, and had a higher level of red blood cell (RBC), lower level of hemoglobin, albumin, eGFR, high‐density lipoprotein (HDL) (*P* < .05); those with HR risers had a higher possibility of CV history, using ß blocker, and a higher level of RBC, and lower level of hemoglobin, albumin (*P* < .05) (Table 1). As shown in Table 2, higher level of night SBP, HR, SBP/ DBP dippers, and poorer ABP control was found in patients with HR non‐dippers or risers at night (Table 2). There were no differences among the three groups in terms of gender, BMI, DM, creatinine, subclinical cardiac damage, including, urinary albumin‐ creatinine ratio (UACR) (Table 1).

**TABLE 1:** Baseline characteristics grouped by HR dipping patterns

Variables	HR dippers (No. = 108)	HR non‐dippers (No. = 152)	HR risers (No. = 42)
Age (years)	69 (62‐78)	76 (67.75‐81)*	75 (67.5‐81.25)
Gender (male, no., %)	50 (46.3)	67 (44.1)	16 (38.1)
BMI (kg/m^2^)	24.4 (22.72‐26.54)	23.73 (22.40‐27.10)	23.73 (20.89‐26.38)
Smoking (no., %)	20 (18.5)	22 (14.5)	6 (14.3)
Alcohol (no., %)	19 (17.6)	22 (14.5)	4 (9.5)
CV history (no., %)	31 (28.7)	65 (42.8)	26 (61.9)*
Arrythmia (no., %)	27 (25)	46 (30.3)	9 (21.4)
Diabetes mellitus (no., %)	78 (72.2)	108 (71.1)	32 (76.2)
Calcium channel blocker (no., %)	71 (65.7)	91 (59.9)	25 (59.5)
Renin angiotensin system (no., %)	50 (46.3)	77 (50.7)	24 (57.1)
Diuretic (no., %)	11 (10.2)	21 (13.8)	8 (19.0)
ß blocker (no., %)	27 (25.0)	47 (30.9)	21 (50.0)*
α blocker (no., %)	1 (0.9)	3 (2)	1 (2.4)
Numbers of drugs (> 2) (no., %)	17 (15.7)	29 (19.1%)	10 (23.8)
Red blood cells (10^12^/L)	4.32 (0.71)	4.06 (0.70)*	3.96 (0.60)*
Hemoglobin (g/L)	127.81 (20.30)	120.14 (20.52)*	117.12 (19.67)*
White blood cells (10^9^/L)	6.15 (5‐7.27)	6.16 (4.83‐7.62)	6.16 (4.73‐8.24)
Albumin (g/L)	40.8 (38.1‐43.4)	38.75 (35.6‐41.43)*	38.45 (34.82‐40.82)*
Creatinine (μmol/L)	107.5 (88.95‐124.4)	113.1 (92.23‐146.34)	104.85 (90.45‐131.27)
Uric acid (μmol/L)	335.5 (277‐436.5)	366 (288.75‐461.25)	368.5 (298‐430.25)
eGFR (mL/min per 1.73 m^2^)	52.98 (44.2‐63.85)	48.15 (35.89‐59.79)*	49.12 (37.01‐56.48)
Triglyceride (mmol/L)	1.47 (0.92‐2.69)	1.37 (0.89‐2.23)	1.14 (0.81‐1.90)
High density lipoprotein (mmol/L)	1.25 (1.02‐1.57)	1.1 (0.92‐1.42)*	1.15 (0.93‐1.52)
Low density lipoprotein (mmol/L)	2.85 (2.26‐3.44)	2.66 (2.02‐3.50)	2.83 (2.21‐3.71)
UACR	91.06 (37.21‐227.43)	98.85 (36.35‐260.26)	154.97 (48.67‐397.16)

* *P* < .05 vs HR dippers.

Abbreviations: BMI, body mass index; CV, cardiovascular; eGFR, estimated glomerular filtration rate; UACR, urine albumin creatine ratio.

**TABLE 2:** The characteristics of ABPM grouped by HR dipping patterns

Variables	HR dippers (no. = 108)	HR non‐dippers (no. = 152)	HR risers (no. = 42)
Office SBP (mmHg)	140 (130‐155)	140 (130‐150)	140 (130‐160)
Office DBP (mmHg)	80 (70‐90)	80 (70‐87)	80 (70‐82)
Office HR (bpm/min)	80 (74‐87)	78 (68‐87)	78 (73‐93)
24h			
SBP (mmHg)	126 (115‐134)	127 (117‐140)	127 (115‐142)
DBP (mmHg)	67 (62‐74)	65 (59‐71)	65 (57‐73)
HR (bpm/min)	72 (67‐80)	71 (65‐78)	70 (64‐80)
Day			
SBP (mmHg)	127 (116‐137)	128 (118‐140)	129 (114‐142)
DBP (mmHg)	68 (62‐75)	65 (59‐71)*	65 (57‐71)
HR (bpm/min)	74 (69‐82)	72 (66‐79)*	69 (63‐79)*
Night			
SBP (mmHg)	121 (112‐132)	129 (116‐139) *	129 (121‐144)*
DBP (mmHg)	64 (58‐69)	63 (57‐70)	65 (58‐78)
HR (bpm/min)	64 (59‐69)	68 (62‐75)*	72 (66‐84)* ^,^ ^†^
SBP dippers	0.96 (0.91‐1)	0.99 (0.95‐1.04)*	1.01 (0.98‐1.07)*
DBP dippers	0.93 (0.89‐0.97)	0.98 (0.92‐1.03)*	1.02 (0.97‐1.09)* ^,^ ^†^
Office BP control (no., %)	41 (38.0)	66 (43.4)	21 (50.0)
ABP control (no., %)	48 (44.4)	47 (30.9)*	10 (23.8)*

* *P* < .05 vs HR dippers.

^†^ *P* < .05 vs HR non‐dippers.

Abbreviations: SBP, systolic blood pressure; DBP, diastolic blood pressure; HR, heart rate; BP, blood pressure; ABP, ambulatory blood pressure.

### 3.2 Renal outcomes

Over a mean follow‐up of 34 ± 17 months, a total of 34 (11.3%) patients reached renal endpoint (25 patients with eGFR decline ≥50%, 24 with progression to ESRD), 56 (18.5%) patients had an eGFR decline ≥30%.

**FIGURE 1:**
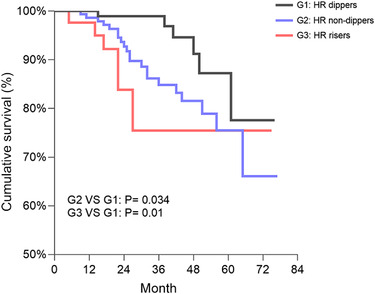
Kaplan‐Meier survival estimates for CKD progression by different HR dipping pattern groups. Abbreviations: CKD, chronic kidney disease; HR, heart rate

A higher proportion of reaching renal endpoint was associated with HR non‐dippers (13.8%) and HR risers (16.7%), versus HR dippers (5.6%) (*P* < .05); cumulative survival of CKD progression was significantly lower in patients with HR non‐dippers (*P* = .034) and HR risers (*P* = .01) when compared to HR dippers (shown in Figure 1).

In the Cox regression model, both HR non‐dippers (hazard ratio 2.58, 95% CI 1.04–6.4, *P* < .05) and HR risers at night (hazard ratio 3.95, 95% CI 1.33–11.79, *P* < .05) were demonstrated to be associated with the renal endpoint, even after adjustment of basic clinical characteristics (arrythmia, use of β blocker, use of calcium channel blocker) (Table 3); however, after further adjustment, HR non‐dippers was not predictive to the renal endpoint, while HR risers could still strongly predict the renal endpoint, independent of baseline eGFR, 24h HR and BP dipping (Table 3).

**TABLE 3:** The association of HR dipping patterns with renal endpoint

Variables	Model 1	Model 2	Model3
HR dippers	Reference	Reference	Reference
HR non‐dippers	2.58 (1.04, 6.40)*	2.56 (1.03, 6.39)*	1.24 (0.47, 3.31)
HR risers	3.95 (1.33, 11.79)*	4.91 (1.59, 15.09)^†^	3.28 (1.03, 10.48)*

* *P* < .05.

^†^ *P* < .01.

Model 1: unadjusted.

Model 2: model 1+arrythmia, use of β blocker, use of calcium channel blocker.

Model 3: model 2+eGFR, 24h heart rate, systolic blood pressure dippers.

In the further research, both HR non dippers (hazard ratio 2.15, 95% CI 1.13–4.09, *P* < .05) and risers (hazard ratio 2.65, 95% CI 1.14–6.14, *P* < .05) showed strong predictive value to a composite renal outcome of ESRD or eGFR decline ≥30% (Table 4). When ESRD, eGFR decline ≥30% or 50% were analyzed separately, HR risers was shown to be an important risk factor for both eGFR decline ≥50% (hazard ratio 5.28, 95% CI 1.45–19.16, *P* < .05), and eGFR decline ≥30% (hazard ratio 2.59, 95% CI 1.07–6.28, *P* < .05); while HR non‐dippers only showed association with eGFR decline ≥30% (hazard ratio 2.13, 95% CI 1.09–4.24, *P* < .05) (Table 4). No associations of HR dipping patterns with ESRD were found.

**TABLE 4:** The association of HR dipping patterns with secondary outcomes

Outcomes	HR dippers	HR non‐dippers (Hazard ratio, 95% CI)	HR risers (Hazard ratio, 95% CI)
Decline in eGFR≥30%, or ESRD	Reference	2.15 (1.13, 4.09)*	2.65 (1.14, 6.14)*
Decline in eGFR ≥ 50%,	Reference	2.83 (0.97, 8.25)	5.28 (1.45, 19.16)*
Decline in eGFR ≥ 30%,	Reference	2.13 (1.09, 4.24)*	2.59 (1.07, 6.28)*
ESRD	Reference	4.41 (0.93, 20.88)	5.26 (0.57, 48.74)
CV events	Reference	0.67 (0.43, 1.06)	0.71 (0.36, 1.43)
Death	Reference	0.74 (0.29, 1.88)	1.49 (0.43, 5.25)

* *P* < .05.

Note: Results were estimated from Cox regression model, adjusted by gender, age, smoking, alcohol, arrythmia, use of calcium channel blockers, use of β blockers, baseline eGFR.

**FIGURE 2:**
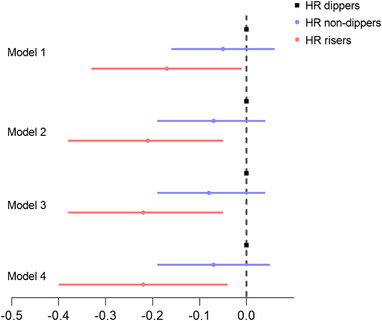
Association of HR dipping patterns with change rate of eGFR from baseline in the multiple linear regression model. *P < .05; **P < .01; Abbreviations: eGFR, estimated glomerular filtration rate; HR, heart rate. Model 1: unadjusted. Model 2: model 1+age, sex, alcohol, smoking, arrythmia, use of calcium channel blocker, use of β blocker. Model 3: model 2+eGFR. Model 4: model 3+ day heart rate, day SBP, night SBP, SBP dippers, DBP dippers

When we evaluated the change of kidney function by eGFR, the eGFR in HR dippers and HR non‐dippers group did not decrease while that in HR risers group decreased obviously (from 49.12 at baseline to 44.5 mL/min/1.73 m^2^ at endpoint); the mean relative change rate of eGFR from baseline was 15%, 11%, and −1.69% in HR dippers, HR non dippers, and HR risers group, respectively. In the multivariate linear regression, HR risers was demonstrated to be negatively correlated with the relative change rate of eGFR from baseline (ß coefficient −0.17, 95% CI ‐0.33‐ ‐0.01, *P* < .05) compared to HR dippers, the difference remained significant after adjustment of day HR, BP and SBP/DBP dipping pattern (shown in Figure 2). Conversely, HR non‐dippers were not shown to be correlated with the relative change rate of eGFR (shown in Figure 2).

### 3.3 CV events and death

During the follow‐up time, there are 8 (7.4%), 11 (7.2%), and 4 (9.5%) death in the HR dippers, HR non‐dippers, and HR risers group; 37 (34.3%), 47 (30.9%) and 11 (26.2%) patients occurred new CV complications in the three groups, respectively. However, the difference in the incidence rate of death and CV events among the three groups was not significant in the analysis of the log‐rank test. In the Cox regression model, the HR non‐dipping pattern was not predictive of death and CV events (Table 4, *P* > .05).

## 4 DISCUSSION

In this two‐center prospective longitudinal study, we reported that the prevalence of HR non‐dipping pattern in CKD 1–4 stage patients with hypertension was 64.2%, and firstly demonstrated that HR non‐dipping pattern, especially the HR risers, could predict effectively renal endpoint and the deterioration of kidney function, independent of BP dipping pattern and 24h HR in these patients; when evaluating change in kidney function by the relative change rate of eGFR from baseline, HR risers were demonstrated to be negatively associated with the change in eGFR, indicating that nocturnal HR rising from the day level was correlated with more declined eGFR. These data suggest that HR non‐dipping pattern, especially HR rising pattern, plays a vitally important role in poot renal outcomes in hypertensive patients with CKD.

Due to the circadian rhythm of the autonomic nervous system, the parasympathetic nervous system is normally dominant at night, causing HR decline.^8^, ^25^ Abnormal variation of HR rhythm has very complex pathogenesis, including abnormal circadian gene expression,^26^, ^27^ and abnormal neurohumoral system regulation, etc.^28^ This will lead to the increment of sympathetic excitability, nocturnal HR elevates consequently, causing a non‐dipping pattern of HR. The prevalence of HR non‐dipping pattern in our study is relatively high, with the rate of 64.2%. For the reason, the patients we enrolled were those who had both CKD and hypertension, which indicated they might originally have an imbalance of autonomic nervous system resulting in relative dominance of sympathetic nervous system,^9^ then the high incidence of HR non‐dippers in these participants could be explained.

HR non‐dipping pattern have been proved to have effects on physiological and pathological processes of heart and kidney, resulting in preclinical cardiac damage, microalbuminuria, CV complications and even all‐cause or CV mortality, as previous studies reported.^8^, ^14^, ^17^, ^29^ However, studies on the association of HR dipping pattern with prognosis in CKD patients are very limited. The first study related to the HR dipping pattern in CKD patients occurred in 2019,^19^ which reported the incidence of non‐dipping HR pattern was higher in hypertensive patients with CKD than that in hypertensive patients without CKD; furthermore, the risk of non‐dipping HR increased with the severity of renal insufficiency.^19^ As our study illustrated, HR non‐dipping pattern accelerated the CKD progression and resulted in a poor renal prognosis, so it is essentially a vicious circle and alerts us of the importance and necessity of paying attention to monitoring circadian rhythms of HR in hypertensive patients with CKD. Despite pieces of evidences on the clinical usefulness of nocturnal HR monitor in predicting renal function impairment and cardiac damage, the current guidelines do not consider the HR monitor in the therapeutic management of CKD,^30^, ^31^ circadian rhythm of HR deserves further attention in CKD patients.

Notably, in our study, HR risers were demonstrated to be more associated with poor renal outcomes compared to HR non‐dippers. Similar findings can be seen in BP, as some previous studies reported, HR risers were correlated with worse renal function than non‐dippers and dippers.^32^, ^33^ Our previous study also identified that high prevalence of nocturnal hypertension (65%) and non‐dipping BP pattern (90.5%) was associated with CKD progression^34^; Considering the consistency of mechanism of nocturnal rising BP and HR, it is not surprising that HR risers were found to have a stronger association with poor renal outcomes in this study. HR rising signifies an extreme variation in circadian HR rhythm, and a more increment of sympathetic tone than non‐dippers and dippers,^35^ which is often considered as an especially harmful HR phenotype, the same as BP rising.^36^ However, there were no studies underlining the predictive value of HR rising pattern previously, our findings need further validation.

In our study, we concluded that HR risers were negatively correlated with the relative change rate of eGFR from baseline (ß coefficient ‐0.17, 95% CI ‐0.33‐ ‐0.01, *P* < .05) whereas HR non‐dippers had no correlation with change rate of eGFR compared to HR dippers (Figure 2). The mean eGFR in HR risers group decreased by 1.69% after follow‐up, while those in HR dippers and HR non‐dippers groups increased by 15% and 11%, respectively, with no change of CKD stages. For the reason, it could be explained that HR rising might have more negative effects on renal function than HR dippers and HR non‐dippers, resulting in the decreased change of eGFR. The level of eGFR in HR dippers and non‐dippers increased slightly, this may be attributed to some other factors, such as drugs (angiotensin‐converting enzyme inhibitor (ACEI) or angiotonin receptor blocker (ARB)) or the improvement of renal vessels perfusion. Some other reasons need to be explored and more studies with large sample size are still acquired.

In the study, we also analyzed the association of HR dipping pattern with CV events. It is not established whether HR non‐dipping pattern can be risk factors of CV events, in contrast to the established relationship of non‐dippers of BP. A previous study of 2021 participants concluded that a non‐dipping pattern of HR was associated with preclinical cardiac damage and predictive to CV events (hazard ratio 1.8, 95% CI: 1.13–2.86, *P* < .01).^17^ Some researchers also drew the same conclusions,^29^, ^37^ while some others not.^14^, ^38^ Our findings are consistent with the latter, no associations were found between HR non‐dipping pattern and CV complications. Reasonable explanations for the contradiction may be the differences in the study population, the previous studies mainly enrolled general or hypertensive patients without CKD while our study included patients with hypertension and CKD; in addition, the small sample size and relatively short follow‐up time of our study may be the other reasons. Prospective well‐designed researches with enough sample size are required.

There are some strengths of our study. Firstly, previous published studies have mostly focused on the association of BP rather than HR dipping pattern with the renal and heart outcomes in CKD patients^33^, ^39^; the researches on the abnormal circadian rhythm of HR has mainly limited to the general or hypertensive patients without CKD,^8^, ^17^, ^29^ our study provides the first evidence on the predictive value of HR dipping pattern to renal and heart outcomes in hypertensive patients with CKD, which may provide a great basis for clinical practice to monitor and manage HR for those CKD patients. Secondly, patients have comprehensive 24‐h ambulatory HR data and clinical assessments; furthermore, our research had a very low rate of lost to follow‐up (0.66%). These strengths make our research result more reliable.

Our study also has some limitations. First, with only 302 patients included into the study, the sample size is relatively small; second, the median follow‐up time is also not long enough with only 34‐month. Further prospective studies with larger sample sizes and long follow‐up periods are needed in the future.

## 5 CONCLUSIONS

Nocturnal HR non‐dipping pattern, especially HR rising, was associated with increased risk of renal endpoint, kidney function deterioration and decline in eGFR. The predictive value of HR non‐dippers for poor renal outcomes disappeared after further adjustment, while that of HR risers remained stable. Circadian rhythm of HR in hypertensive patients with CKD deserves further attention and research.

## CONFLICT OF INTEREST

The authors declared no potential conflicts of interest concerning the research, authorship, and/ or publication of this article.

## AUTHOR CONTRIBUTIONS

Xiang Liu, Huan Zhou, Yi Tang, and Wei Qin: Substantial contributions to the conception or design of the work; or the acquisition, analysis, or interpretation of data for the work; AND drafting the work or revising it critically for important intellectual content; AND final approval of the version to be published; AND agreement to be accountable for all aspects of the work in ensuring that questions related to the accuracy or integrity of any part of the work are appropriately investigated and resolved.

Gen Li, Fangming Li, Lingqiu Dong, and Siqing Wang, Zheng Jiang, Jiaxing Tan, Aiya Qin: analysis and acquisition of data; AND drafting the work; AND final approval of the version to be published; AND agreement to be accountable for all aspects of the work in ensuring that questions related to the accuracy or integrity of any part of the work are appropriately investigated and resolved.
